# Right Ventricle Thrombus in a Massive Pulmonary Embolism COVID-19 Patient

**DOI:** 10.7759/cureus.43937

**Published:** 2023-08-22

**Authors:** Vasco Gaspar, Bernardo Silva, Inês Ambrioso, Cláudia Alves, Martim Alçada

**Affiliations:** 1 Internal Medicine Department, Hospital Distrital de Santarém, Santarém, PRT

**Keywords:** pulmonary embolism (pe), intracardiac thrombus, obstructive shock, pulmonary infarction, covid-19 pneumonia, cardiogenic shock, massive pulmonary embolism, right ventricle thrombus, covid-19

## Abstract

The coronavirus disease 2019 (COVID-19) infection presents with a wild range of clinical manifestations. Increased inflammatory response and thrombotic risk have been described, being pulmonary embolism a potential cause of death in these patients. Pulmonary embolisms with right ventricle thrombus are rare and have higher mortality rates. This case report concerns a rare clinical presentation of a 75-year-old male with a medical history of right renal transplantation 36 years ago, that presented with a ten-day history of asthenia, followed by fever, shortness of breath, and cough since the day before. He was admitted with severe acute respiratory syndrome coronavirus-2 (SARS-CoV-2) pneumonia and respiratory insufficiency. The next morning the patient worsened, he presented with hypotension, tachycardia, severe refractory hypoxemia, and chest pain. Contrast CT showed a massive pulmonary embolism with a right ventricle thrombus, confirmed by an echocardiogram. Anticoagulation and IV fluids were started, and the patient was transferred to the ICU. He developed obstructive shock, so thrombolysis was performed with a full dose of alteplase. The outcome was good with complete recovery. Posterior investigation excluded other causes for pulmonary embolism. The severity of pulmonary parenchymal disease secondary to COVID-19 correlates with thromboembolic complications, which demand a swift response to avoid death. An abrupt deterioration in oxygenation should raise suspicion for PE in COVID-19 patients, and mostly in the presence of hypotension and tachycardia. In our case report, there was a massive pulmonary embolism with a rare right ventricle thrombus that had a good outcome with medical treatment.

## Introduction

The coronavirus disease 2019 (COVID-19) pandemic caused by the severe acute respiratory syndrome coronavirus-2 (SARS-CoV-2) virus presents with a wide range of clinical manifestations [1]. The typical presentation of severe infection is viral pneumonia with respiratory insufficiency. It can also affect multiple systems, including the cardiovascular system [2,3].

The SARS-CoV-2 virus enters the cells via angiotensin conversion enzyme-2 (ACE-2), which is wildly expressed in the heart, vessels, and lungs [2,4]. The inflammatory response caused by the virus induces endothelial dysfunction and is responsible for a hypercoagulable state that causes thrombotic complications [2,3,5]. Elevated levels of D-dimer, and other inflammatory markers, have been associated with the inflammatory response and thrombotic risk [5]. These thrombotic complications can present from silent myocardial injury to acute coronary syndromes, arrhythmia, stroke, venous thromboembolism, pulmonary embolism (PE), intracardiac thrombi, and cardiogenic shock. In most cases, the proinflammatory and prothrombotic conditions correlate with the severity of the COVID-19 infection and hence the risk increases with severe COVID-19 pneumonia [2,3,6-13].

PE is one of the most severe thrombotic complications of COVID-19 [2]. The overall incidence of PE in COVID-19 varies between 0.4% and 3.4% but rises to 17%-27% in critical patients [2,14-16]. Cardiac thrombi are rare but have been described [6-13,17]. Right heart thrombi are detected in approximately 4% of patients with PE, and they are associated with higher mortality rates [17]. It has been reported that PE was the direct cause of death in one-third of COVID-19 patients' autopsies [18].

An abrupt deterioration in oxygenation should raise suspicion for PE in COVID-19 patients, and mostly in the presence of hypotension and tachycardia [2,3]. The early diagnosis of PE and prompt treatment are vital [2]. The treatment of intracardiac thrombi can be with either anticoagulation, fibrinolytic therapy, and/or surgical embolectomy depending on the stability of the patient as well as the balance of its risks and benefits [17,19].

## Case presentation

The patient was a 75-year-old male that presented to the emergency with a ten-day history of asthenia followed by fever, shortness of breath, and cough since the day before. His past medical history included right renal transplantation 36 years ago, with no complications and regular use of prednisolone and azathioprine. The vital signs showed tachycardia (HR 100 bpm), normal blood pressure (BP 100/67 mmHg), apirexia (TT 36.4 ºC), and low O_2_ saturation (91% in a 28% Venturi mask) with a respiratory rate of 14 breaths per minute. Physical examination revealed bilateral rales and the use of accessory muscles. Chest x-ray revealed a normal cardiac silhouette and evidence of bilateral pneumonia. Chest CT showed extensive bilateral pneumonia with ground-glass opacities and consolidation and crazy-paving pattern. Laboratory investigation revealed an elevated C-reactive protein at 156 mg/L (normal range <6 mg/L), elevated white blood cells of 11.6 x 10^9^/L (normal range 4 - 10 x10^9^/L) with prominent neutrophilia (78%); hemoglobin was normal (14 g/dL) as well as procalcitonin (0.15 ng/mL) and renal function (creatinine 1 mg/dL, urea 53 mg/dL); it also revealed an elevated high sensitivity troponin I of 2,397 ng/L (normal range < 14 ng/L), an N-terminal pro-brain natriuretic peptide (NT-proBNP) of 13,287 pg/mL (normal range <100 pg/mL). The SARS-CoV-2 PCR test was positive. Arterial blood gas (FiO_2_ 28%) showed type I respiratory insufficiency (pH 7.48, PaCO_2_ 27 mmHg, PaO_2_ 68 mmHg, HCO_3_ 24 mmHg, lactate 1.3 mmol/L). An electrocardiogram (ECG) showed sinus tachycardia, with right brunch block, without evidence of ischemia. Urine antigen tests for Legionella pneumophila and Streptococcus pneumoniae were negative. Blood cultures were also negative.

**Figure 1:**
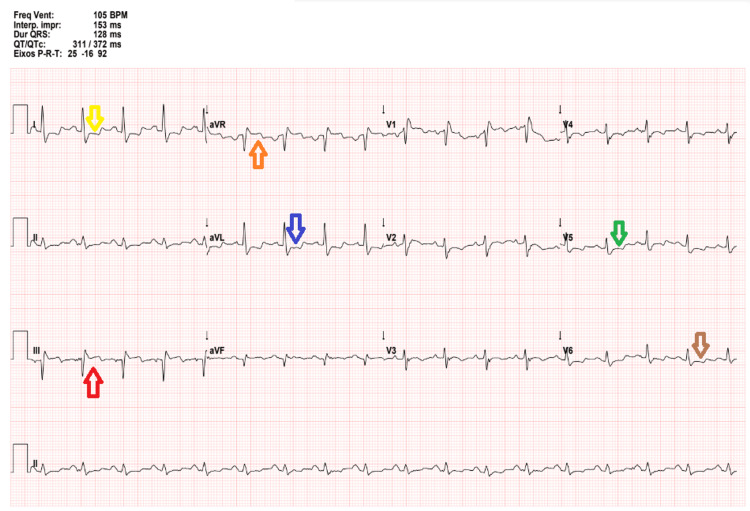
Electrocardiogram with sinus tachycardia, right brunch block, inframillimetric ST elevation in AVR (orange arrow) and DIII (red arrow), and inframillimetric ST depression in DI (yellow arrow), AVL (blue arrow), V5 (green arrow) and V6 (brown arrow)

The patient was admitted to the COVID-19 ward and started on dexamethasone and IV ceftriaxone and azithromycin for COVID-19 pneumonia with suspected secondary bacterial infection. The next morning patient deteriorated with hypotension (BP 89/53 mmHg), tachycardia (HR 110 bpm), increased respiratory rate (18 breaths per minute) with the use of accessory muscles, shortness of breath while speaking, and desaturation. He also complained of sharp and stabbing type chest pain 8/10, that radiated to the back. The patient was hypoxic so non-rebreather mask at 15 L/min was started. ECG showed sinus tachycardia, with right bundle branch block, with inframillimetric ST elevation in AVR and DIII, and inframillimetric ST depression in DI, AVL, V5, and V6 (Figure 1). Blood arterial gas (FiO_2_ 60%) showed respiratory alkalemia with hypoxemia and (pH 7.49, PaCO_2_ 23 mmHg, PaO_2_ 75 mmHg, HCO_3_ 21 mmHg, lactate 2.2 mmol/L). Anticoagulation with low-molecular-weight heparin (LMWH) and IV fluids was started.

**Figure 2:**
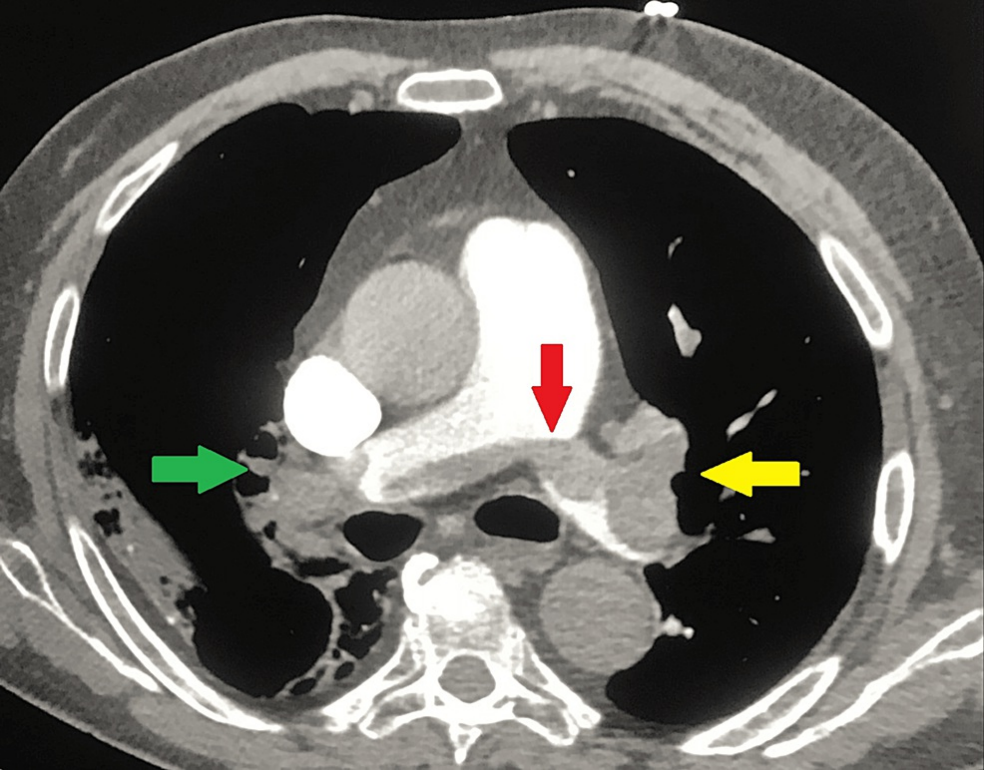
CT pulmonary angiography showing massive pulmonary embolism CT pulmonary angiography showing a red arrow pointing at massive pulmonary embolism communicating with both main pulmonary arteries, a yellow arrow pointing at the occlusion of left lobar arteries, and a green arrow pointing at the occlusion of right lobar arteries.

**Figure 3:**
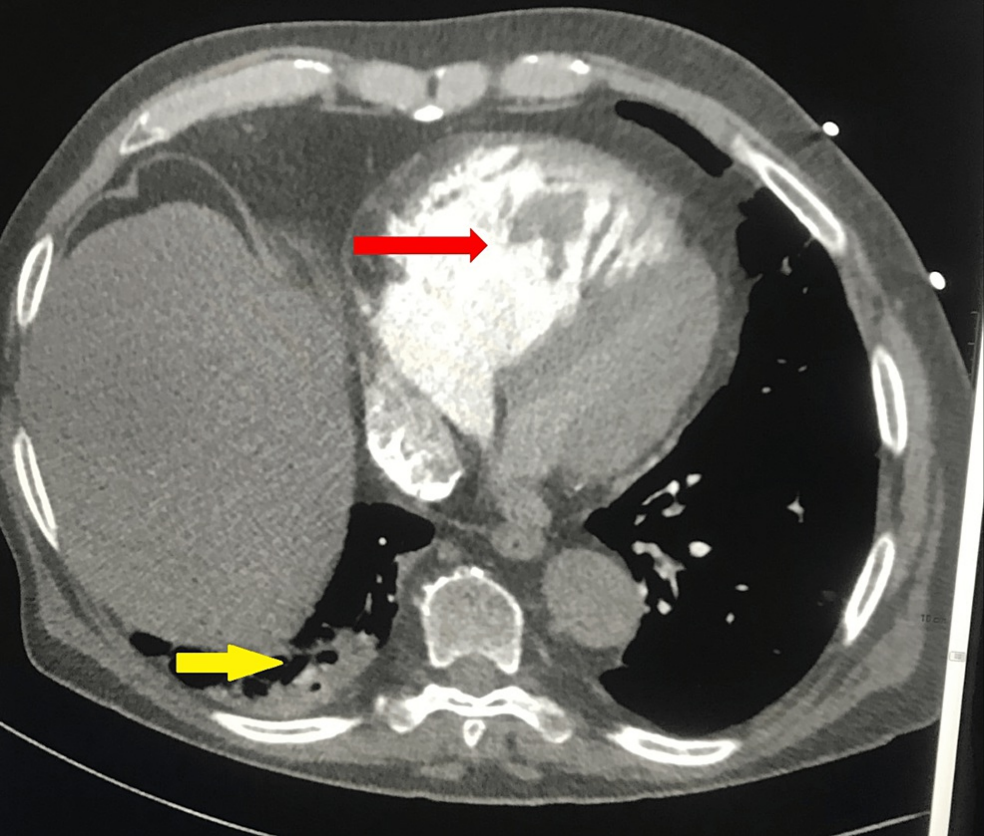
CT showing a red arrow pointing at the thrombus in the right ventricle and a yellow arrow pointing at consolidation in the right inferior lobe

Contrast CT showed a massive PE with thrombosis of all pulmonary arteries and communication between the two principal arteries (Figure 2), a right ventricle (RV) thrombus was noticed (Figure 3), as well as the previously known extensive bilateral extensive infiltrates. Bed-sided transthoracic echocardiography (TTE) showed RV dilation with positive Mc Connell sign, but normal systolic longitudinal function (TAPSE 21 mm), RV large mobile apical mass with no relation to the valve system, D-shaped left ventricle with preserved systolic function. Blood work showed elevated D-dimers of 38,336 ng/mL (normal range < 500 ng/mL), compared to the day before C-reactive protein raised to 232 mg/L, and high sensitivity troponin I decreased to 1,103 ng/L.

The patient was transferred to the intensive care unit (ICU). The patient worsened a few hours later with refractory hypotension and respiratory difficulty despite proper oxygenation, so it was admitted that the PE was causing obstructive shock. Thrombolysis was performed with full dose alteplase (100mg) for two hours, followed by non-fractionated heparin (NFH) in the dose of 18 U/kg/h for three days and posterior switch to LMVH. A venous Doppler ruled out deep vein thrombosis. The ICU stay was complicated with a minor hematoma of the right superior arm with no vascular or neurologic compromise, nor hemodynamic instability or even significant hemoglobin decrease. The control TTE in the fourth day of admission showed improvement and the RV thrombus was no longer detected. Ceftriaxone and dexamethasone were maintained for eight days and azithromycin for five days with reduction of the inflammatory markers. Clinical improvement allowed oxygen reduction and step-down of care and anticoagulation was switched to apixaban.

The patient was discharged with a normal TTE and reevaluated in outpatient clinic. Malignancy was ruled out by complete body CT and upper and lower gastrointestinal endoscopy. Hypercoagulable state was also ruled out with negative anticardiolipin, antinuclear, antiphospholipid, and beta-2-glycoprotein antibodies, as well as normal homocysteine, antithrombin, protein C and protein S. Anticoagulation was maintained for six months. No other thrombotic events were registered. The COVID-19 infection appeared to be the cause of the RV thrombus and massive PE.

## Discussion

Though affecting mostly, the respiratory system, causing severe pneumonia and even acute respiratory distress syndrome (ARDS) [1]. COVID-19 infection may cause hypercoagulopathy and thrombotic complications that have been well-accessed in the literature [2,4,5].

McFadyen et al. supported this by showing that the thrombotic manifestations of severe COVID-19 are related to the endothelial dysfunction caused by the SARS-CoV-2 ability to directly infect endothelial cells via ACE-2 receptors expressed on the endothelial cell surface in the lungs, vessels, and heart [4]. Trepa et al. reviewed as PE is one of the most severe thrombotic complications of COVID-19, and that its incidence is likely higher than other known viral infections. The incidence varies in the literature, but there is a consensus that is higher in critical patients [2,10,14,16].

Ogren et al. studied the prevalence and risk of PE in patients with intracardiac thrombosis [11]. As Sakellariou et al. reviewed right heart thrombi are detected in approximately 4% of patients with PE [17]. RV thrombus is even rarer than right atria, but both have been described [6-13]. The treatment of intracardiac varies with each case, each patient, their hemodynamic stability, and the balance of their risks and benefits. The treatment includes anticoagulation, fibrinolytic therapy, and/or surgical embolectomy [17,19].

Sakellariou et al. reviewed that mobile right heart thrombi require rapid therapeutic choices between surgical thrombectomy and thrombolysis. They also presented a successful case of medical thrombolysis [4]. The absence of cardiothoracic surgery service in our hospital, as well as the high distance to a center where thrombectomy was feasible, ruled out that option. The fast deterioration of the patient's status with evolution to cardiac shock demanded fast action, and the prompt start of fibrinolytic therapy with a full dose of alteplase was successful.

Wichmann et al. established that PE was the direct cause of death in a third of COVID-19 patients that were autopsied [18]. Tang et al. associated raised d-dimers with severe presentation and death [5]. Despite these risk factors and also the increased risk associated with the presence of intracardiac thrombus, our patient had a good outcome.

Klok et al. and Trepa et al. both explored the importance of prevention with thrombosis prophylaxis in COVID-19 as well as the vital importance of monitoring these patients for signs of thrombotic complications [2,3]. In our case, the suspicion of PE should have been made at admission, especially on the presence of tachycardia, and elevation of troponin and BNP, despite the uncharacteristic initial ECG. D-dimers should have been tested to rule out PE, the diagnosis would probably have been made some hours earlier.

In the present case, the patient was admitted at the end of 2020, to a secondary hospital in Portugal, at the peak second wave of COVID-19. The available resources were scarce, both human and material resources (echocardiogram was not usually available outside working hours, and its use on COVID-19 patients was very limited; hemodialysis was also not available for COVID-19 patients), and supply shortages of medical resources were even more exacerbated by disruptions to the global supply chain [20,21]. Nevertheless, reviewing the case, this patient should have had a contrast CT at admission.

## Conclusions

Hypercoagulability is a known complication of COVID-19 and thrombosis can occur in various forms, including PE and RV thrombus. These complications are more frequent in critically ill patients, and their severity is linked with the severity of COVID-19 pneumonia. Despite being potentially fatal, a massive PE with an RV thrombus can be successfully treated with medical treatment.

## References

[1] Huang C, Wang Y, Li X (2020). Clinical features of patients infected with 2019 novel coronavirus in Wuhan, China. Lancet.

[2] Trêpa MA, Hipólito Reis A, Oliveira M (2021). Cardiovascular complications of COVID-19 infection. Acta Med Port.

[3] Klok FA, Kruip MJ, van der Meer NJ (2020). Incidence of thrombotic complications in critically ill ICU patients with COVID-19. Thromb Res.

[4] McFadyen JD, Stevens H, Peter K (2020). The emerging threat of (micro)thrombosis in COVID-19 and its therapeutic implications. Circ Res.

[5] Tang N, Li D, Wang X, Sun Z (2020). Abnormal coagulation parameters are associated with poor prognosis in patients with novel coronavirus pneumonia. J Thromb Haemost.

[6] Terrigno VR, Tan JL, Singh D, Sabir SA (2020). Right atrial thrombus in a patient with COVID-19. Cureus.

[7] Shamsah MA, Bitar ZI, Alfoudri H (2020). Right atrial thrombus in a patient with COVID-19 pneumonia: a case report. Eur Heart J Case Rep.

[8] Khalil F, Hamza Saad Shaukat M, Perinkulam Sathyanarayanan S, Stys A (2022). Post COVID-19 large atrial thrombus found incidentally. S D Med.

[9] Mitsis A, Alexi A, Constantinides T, Chatzantonis G, Avraamides P (2022). A case of right ventricular thrombus in a patient with recent COVID-19 infection. Cureus.

[10] Benjamin MM, Afzal A, Chamogeorgakis T, Feghali GA (2017). Right atrial thrombus and its causes, complications, and therapy. Proc (Bayl Univ Med Cent).

[11] Ogren M, Bergqvist D, Eriksson H, Lindblad B, Sternby NH (2005). Prevalence and risk of pulmonary embolism in patients with intracardiac thrombosis: a population-based study of 23 796 consecutive autopsies. Eur Heart J.

[12] Lai E, Alishetti S, Wong JM, Delic L, Egrie G, Rosenblatt A (2019). Right ventricular thrombus in transit: raising the stakes in the management of pulmonary embolism. CASE (Phila).

[13] Barbagallo M, Naef D, Köpfli P (2021). Right ventricular thrombus, a challenge in imaging diagnostics: a case series. Eur Heart J Case Rep.

[14] Tang N, Bai H, Chen X, Gong J, Li D, Sun Z (2020). Anticoagulant treatment is associated with decreased mortality in severe coronavirus disease 2019 patients with coagulopathy. J Thromb Haemost.

[15] Miró Ò, Jiménez S, Mebazaa A (2021). Pulmonary embolism in patients with COVID-19: incidence, risk factors, clinical characteristics, and outcome. Eur Heart J.

[16] Poissy J, Goutay J, Caplan M (2020). Pulmonary embolism in patients with COVID- 19: awareness of an increased prevalence. Circulation.

[17] Sakellariou XM, Efstathopoulos A, Stamatis KV, Nikas DN, Kolettis TM (2020). Treatment of mobile right heart thrombi. Eur J Case Rep Intern Med.

[18] Wichmann D, Sperhake JP, Lütgehetmann M (2020). Autopsy findings and venous thromboembolism in patients with COVID- 19: a prospective cohort study. Ann Intern Med.

[19] Arora G, Madisetty D (2023). Right atrial thrombus and submassive pulmonary embolism in a COVID-19-infected patient: a case report. Cureus.

[20] Cardoso J, Castro I, Gaspar V, Esteves C (2022). COVID-19 pneumonia complicated by pneumomediastinum: a case report. Cureus.

[21] Poon YR, Lin YP, Griffiths P, Yong KK, Seah B, Liaw SY (2022). A global overview of healthcare workers' turnover intention amid COVID-19 pandemic: a systematic review with future directions. Hum Resour Health.

